# Effects of academic self-regulation on procrastination, academic stress and anxiety, resilience and academic performance in a sample of Spanish secondary school students

**DOI:** 10.3389/fpsyg.2023.1073529

**Published:** 2023-02-02

**Authors:** Antonio Ragusa, Jeronimo González-Bernal, Ruben Trigueros, Valeria Caggiano, Noelia Navarro, Luis A. Minguez-Minguez, Ana I. Obregón, Carmen Fernandez-Ortega

**Affiliations:** ^1^Department of Psychology, University of Burgos, Burgos, Spain; ^2^Department of Psychology, University of Almeria, Almería, Spain; ^3^Department of Psychology, University of Rome Tor Vergata, Roma, Lazio, Italy

**Keywords:** procrastination, stress, anxiety, resilience, academic performance

## Abstract

**Introduction:**

One of the biggest challenges facing students today is procrastination, which is closely related to stress, anxiety and, in the most severe cases, depression. The aim of the present study is to analyze the influence of academic self-regulation on procrastination, academic anxiety and stress, academic resilience and academic performance.

**Method:**

The sample consisted of 991 high school students aged between 16 and 19 years (*M* = 17.25; SD = 3.45). A structural equation model was used to analyze the relationship between the study variables.

**Results:**

Analyzes showed that academic self-regulation negatively predicted procrastination. In turn, procrastination positively predicted academic stress and anxiety. However, resilience exerted a protective influence by being positively related to academic stress and anxiety. Finally, resilience positively predicted academic performance, whereas stress and anxiety negatively predicted academic performance.

**Conclusion:**

Thus, the importance of generating strategies for students to learn to self-regulate in academic contexts, manage emotions, foster motivation and develop strategies to help them overcome the vicissitudes they face is emphasized.

## Introduction

Post-compulsory secondary education is often experienced by students as a very stressful and demanding period, as it is decisive for their future academic aspirations (Banks and Smyth, 2015; Pascoe et al., 2020). In addition, the content that is usually worked on in this period is more complex than in compulsory secondary school (Govaerts and Grégoire, 2004). This level of coping puts stress on students and limits their academic decision-making, sometimes leading to maladaptive academic behaviors related to avoidance (Deb et al., 2015). In this way, students often display behaviors to voluntarily postpone activities and/or exams, either because others guarantee immediate achievement, inadequate information processing or fear of failure (Ziegler and Opdenakker, 2018). This leads to academic and personal problems as immediate completion of activities and study becomes difficult (Ziegler and Opdenakker, 2018). Therefore, it is key for students throughout their academic life to develop a range of academic strategies that actively contribute toward their learning goals and to be metacognitively aware, planful and strategic (Teng et al., 2021). Thus, the present study aims to analyze the effect of academic self-regulation on procrastination, resilience, academic stress and anxiety, and academic performance.

### Academic self-regulation

The construction of learning responds to an intentional and conscious activity aimed at achieving a meaningful construction for the student who learns, we therefore speak of strategic behavior and, accordingly, of strategic learning. This learning, in order to be such, must be reflective and aimed at the autonomy of the person, self-direction (Trigueros-Ramos et al., 2019). This requires an active process for students to define their learning goals, trying to know, control, regulate their cognitions, motivations and actions to achieve these goals (Inzlicht et al., 2021).

For this self-regulation process to be successful, it is necessary to effectively use certain skills or strategies of metacognition or self-knowledge that allow a certain emotional understanding and cognitive resources used in the learning process, defining and questioning goals, as well as planning and evaluating the teaching process (Panadero and Alonso-Tapia, 2014). Therefore, it would be a multilevel, interactive and self-directed process, whose origin is located at the social level (Boekaerts et al., 2005).

Although numerous studies have focused on the effects of self-regulated learning, finding that it has a positive influence on the ability to learn how to learn (Wibrowski et al., 2017), self-efficacy (Kandemir, 2014) and in addition, students with high self-regulation skills were also found to make greater use of metacognitive and repetition strategies, as opposed to procrastination (Wolters et al., 2017). However, studies that have analyzed the effect of academic self-regulation have mainly been related to positive or negative factors but have not explored the relationship between these variables as mediated by a negative factor.

### Procrastination

Procrastination is a theoretical construct that explains intentional and unjustified procrastination from a behavioral, cognitive and emotional approach. It involves procrastinating one or several activities, either at the beginning, development or conclusion of them and carrying out another or other unnecessary activities that prevent the completion of the original activity (Glick and Orsillo, 2015; Bolivar et al., 2016). Procrastination involves the individual in the presence of a sense of guilt and discomfort for the very fact of delaying and subsequent relief when the pending activity is executed, usually at the ‘last moment’ (Moreta et al., 2018). In the context of education, studies on the prevalence of procrastination indicate that at least 20% of the young population acknowledges having procrastinatory behaviors (Harriott and Ferrari, 1996), with this percentage in Spanish-speaking countries being 14% (Ferrari et al., 2007). Adolescence and young adulthood are the period of greatest susceptibility to procrastinatory behaviors, with the figure being found to skyrocket, reaching up to 95% of adolescents (Steel, 2010) and between 80 and 95% of university students to develop this behavior, as well as the desire to reduce it (Muñoz-Olano and Hurtado-Parrado, 2017; Codina et al., 2020).

Procrastination does not refer to the failure to complete a task, but to the experience of procrastination and last-minute completion, although non-completion may occur (Pardo et al., 2014). This, of course, affects with a diminished sense of well-being. It results in stress, difficulties in following instructions (Milgram et al., 1992), unpunctuality (Pestana et al., 2020), poor school performance (Kim and Seo, 2015), personality problems (Park and Sperling, 2012). However, studies on procrastination in education are mostly descriptive and unidirectional. Therefore, it is necessary to analyze the role of academic procrastination as a mediator of learning processes and to take into account its influence on negative and positive factors focused on students’ learning processes.

### Stress and anxiety

Academic stress and anxiety can be defined as a relationship between the student and the demands of the academic environment, which is perceived by the former as threatening and endangers their well-being (Trigueros et al., 2020a). Perceived demanding demands (e.g., exams, a competitive context, proximity to deadlines.) can affect students’ performance, as well as their physical and mental health, generating an imbalance that leads them to resort to different coping strategies to restore it (Watson and Watson, 2016). Although academic stress and anxiety reaches its peak during the university stage (Peer et al., 2015), it is not exclusive to this period, and also manifests itself in the other stages of schooling. In this sense, studies focused on the post-compulsory stage are still quite scarce compared to the rest of the educational stages (Trigueros et al., 2020b).

Stress and anxiety have been positively associated with depression, poor academic performance (Karaman and Watson, 2017), school dropout (Pascoe et al., 2020) and even sleep quality (Wunsch et al., 2017). Conversely, it has been found that students who respond adaptively to academic stress tend toward self-care, emotional regulation and show more self-efficacy (Watson and Watson, 2016). On the other hand, it is also important to take into account the effect of anxiety in exams or assessment situations, since the emotional reaction triggered in these contexts, characterized by uncertainty and tension, can provoke physiological and behavioral changes (Trigueros et al., 2020b). Thus, the pressure of exams and activities could be considered as potentially threatening as it could interfere with their future academic goals and add pressure from family or social environment (Deb et al., 2015).

### Resilience

In recent years, there has been a growing interest in the study of academic resilience, especially in terms of how to enhance it given its clear advantages in terms of social, academic and personal skills in the face of adverse situations (Allen and Eklund, 2019). In general terms, a resilient student is one who, despite having disadvantageous social or personal conditions, obtains a clearly higher performance than expected, that is, one who is able to adjust adaptively to the demands of the environment (Trigueros et al., 2020c). However, resilience is not a linear construct in which an event causes a personal maladjustment in the individual, without considering other pathways. Moreover, emotions can affect the process of adaptation and coping (Tudor and Spray, 2017). This is essential given the importance of emotions in protecting individuals’ behaviors, and several studies have shown that people value emotions as facilitators of the development of resilience capacity (Ahmed et al., 2018).

Studies related to resilience have shown that this construct is related to academic performance, as a protective factor against negative emotions, motivation and internal well-being. Despite these studies, academic resilience is a recent construct and there are important gaps on how it can be affected by students’ cognitive characteristics and decisions (Cassidy, 2016).

### Objectives and hypotheses

Based on the above, the aim of the present study is to analyze the influence of academic self-regulation on procrastination, academic anxiety and stress, academic resilience and academic performance. These relationships will be analyzed through a structural equation model where the protective influence of resilience against stress, procrastination and anxiety will be observed. The hypothesis put forward in the study is as follows: (H1) academic self-regulation will negatively predict procrastination; (H2) procrastination will positively predict academic stress and anxiety; (H3) academic resilience will negatively predict academic stress and anxiety, on the contrary, it will positively predict academic performance; (H4) academic stress and anxiety will negatively predict academic performance.

## Methods

### Participants

The sample consisted of 991 high school students (563 boys and 428 girls) aged between 16 and 19 years (*M* = 17.25; SD = 3.45). These participants belonged to several public schools in the province of Almeria (Spain), siguiendo un maestro no probabilistico inferencial ya que no todos los centros educativos mostraron su conformidad para que los estudiantes participaran en el estudio. Participation was voluntary, and they did not receive any incentive for their collaboration.

The G*Power programe was used to calculate the sample size and check the statistical power of the study. For all calculations an *α* = 0.05 and a statistical power (1 - *β*) = 0.80 was considered. The algorithm of O’Brien and Shieh (1999) was used.

### Measurements

Academic Self-Regulation. The self-regulated learning questionnaire by Torre (2007) was used. This questionnaire consists of 20 items represented by four factors: active metacognitive awareness (6 items; e.g. “When I start to study I am clear about when and why I should study in one way and when and why I should use a different strategy”), control and verification by the student (7 items; e.g. “When I am studying a subject, I try to identify things and concepts that I do not understand well”), effort and effort (7 items; e.g. “When I am studying a subject, I try to identify things and concepts that I do not understand well”). “When I am studying a subject, I try to identify things and concepts that I do not understand well”), daily effort in completing tasks (4 items; e.g. “After class, at home, I check my notes to make sure that I understand the information and that everything is in order,” and active processing during class (3 items; e.g. “During class, I frequently check if I am understanding what the teacher is explaining”). The scale is Likert-type, with responses ranging from 1 (nothing to do with me) to 5 (I am like that).

The Irrational Procrastination Scale (IPS) by Steel (2010) was used, specifically the Spanish version (Guilera et al., 2018) composed of 9 items with 5 response options on a Likert-type scale (1 = never; 5 = very often) that assess the frequency with which irrational delay in tasks occurs as a result of procrastination (“My life would be better if I had done some activities or tasks earlier”). This tool has adequate psychometric properties (Alpha coefficient above 0.90), indicating adequate internal consistency.

Academic Stress. The Spanish version (Espejo et al., 2011) validated from the Student Stress Inventory (SSI; Fimian et al., 1989) was used. The SSI is composed of a total of 22 items divided into three factors: physiological (6 items), (e.g., “I have itching all over my body”) emotional (10 items) (e.g., “I feel overwhelmed”) and behavioral (6 items) (e.g., “I neglect my friends”) with Likert-type response options ranging from not at all (1) to strongly agree (5). The internal consistency alpha coefficient values found are 0.79 for the emotional manifestations factor, 0.62 for physiological manifestations and 0.66 for behavioral manifestations, so it has an appropriate internal consistency.

Academic Resilience. The Spanish version of Trigueros et al. (2020) was used, validated from the Academic Resilience Scale of Cassidy (2016). The scale consists of 30 items divided into three factors: reflexivity and adaptive help-seeking (9 items; e.g. “I would seek support from my family and friends”), perseverance (14 items; “I would use the situation to motivate myself”; “I would not change my long-term goals and ambitions”) and, finally, negative affect and emotional response (7 items; “I would start to think that my chances of success at university were low”; “I would probably get depressed”). The scale is Likert-type, with responses ranging from 1 (Likely) to 5 (Unlikely). The results of the internal consistency analysis showed reliability values above 0.80.

Academic Anxiety. The Spanish version of Sesé et al. (2010) validated from The Test Anxiety Inventory (Spielberger, 1980) was used. The scale is composed of 30 items, distributed in 4 factors: emotionality (8 items; e.g. “I feel anxious”), worry (10 items; e.g. “I worry about the exam grade”), interference (6 items; e.g. “I think about anything and I get distracted”) and lack of confidence (6 items; e.g. “I trust my ability”). The scale is Likert-type, with responses ranging from 1 (almost never) to 4 (almost always).

Academic Performance. In order to analyze the performance of secondary school students, the average grade of all subjects at the end of the academic year was taken into consideration. In this sense, the grades were distributed as follows: 5 (outstanding), 4 (outstanding), 3 (good), 2 (pass) and 1 (fail).

### Procedure

The present study is endorsed by the scientific bioethics committee of the University of Almeria (Reference: UAL/BIO 213/2020). Once approval was obtained, a member of the research group contacted the management teams of several schools to request their collaboration, explaining the objectives of the study beforehand. Subsequently, those students whose schools agreed to participate in the study were informed of the objectives. They were given an informed consent form to be signed by their parents/legal guardians if they wished to participate in the study, and after this process a total of 991 students took part. The questionnaires were completed by the students first thing in the morning on an individual basis and in writing, emphasizing that the answers would be anonymous and confidential. A member of the research group was present to answer any questions they might have. The questionnaires took around 30 min to complete.

### Data analysis

**Figure 1:**
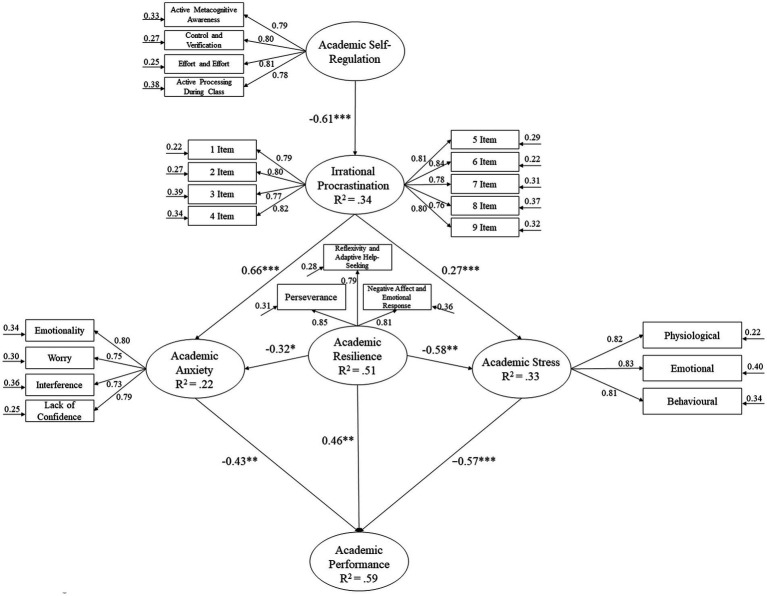
Structural equation model showing the relationships between variables. *p < 0.05; **p < 0.01; ***p < 0.001.

Several analyzes were carried out in this study: descriptive statistical analysis (mean, standard deviation and Pearson’s bivariate correlations), reliability analysis through Cronbach’s alpha and a structural equation model to analyze the predictive relationships established in Figure 1. For the various statistical analyzes carried out in this study, the SPSS v25 and AMOS v20 statistical packages were used.

Prior to SEM analysis, the normality of the data was analyzed using the kurtosis values of the individual variables (Stevens, 2009). Kurtosis values greater than 3.00 may indicate that a variable is not normally distributed (Westfall and Henning, 2013). For the structural equation model, the maximum likelihood method was used, together with a Bootstrapping of 6,000 interactions. The fit indices taken into account for the CFAs and structural equation model were the indices established by Hair Jr et al. (2017) and Hair et al. (2006): χ2/df, with values between 2 and 3; Comparative Fit Index (CFI), Incremental Fit Index (IFI), Tucker Lewis Index (TLI), with values above 0.95; Root Mean Square Error of Approximation (RMSEA, 95% confidence interval), with values below 0.06; Standardized Root Mean Square Residual (SRMR), with values below 0.08. However, according to Marsh et al. (2004), these indices should be taken into account with some caution as they are restrictive if the model is very complex.

## Results

### Preliminary analysis

Table 1 shows the mean, standard deviation and bivariate correlations. The correlations reflected a positive valence between related factors such as self-regulation, resilience and academic performance, and on the other hand between stress, anxiety and procrastination. In contrast, the correlation was negative between the two groups. In addition, Table 2 shows the reliability analyzes with all scores above 0.80 and kurtosis values were not greater than 3.

**Table 1:** Distribution of students by school.

	Boys	Girls	Total
1. High School “*A*”	135	117	252
2. High School “*B*”	147	90	237
3. High School “*C*”	126	111	237
4. High School “*D*”	155	110	265

For ethical considerations, the names of the high schools have not been added.

**Table 2:** Descriptive analyzes, internal consistency, kurtosis and bivariate correlations.

Factors	*M*	SD	*α*	*k*	1	2	3	4	5	6
1. Self-Regulation Academic	3.77	1.33	0.84	1.12		−0.39***	−0.45**	0.31***	−0.52***	0.62***
2. Procrastination	2.45	1.44	0.83	0.78			0.47***	−0.38**	0.51***	−0.42**
3. Academic Anxiety	2.12	1.38	0.82	1.20				−0.68***	0.71***	−0.37***
4. Academic Resilience	4.01	0.77	0.85	0.87					0.75***	0.55***
5. Academic Stress	1.96	1.22	0.80	0.83						−0.54**
6. Academic Performance	2.69	1.07	-	1.55						

*p < 0.05; **p < 0.01; ***p < 0.001. *α* = Cronbach’s Alpha; *k* = Kurtosis.

### Structural equation model

Before testing the hypothesized model, a confirmatory factor analysis was conducted for each of the scales used. The data revealed (Table 3) that each of the scales had acceptable fit indices, taking into account the fit indices described by Hair et al. (2006).

**Table 3:** Confirmatory factor analysis of the scales.

Scales	χ2/df	CFI	IFI	TLI	RMSEA	SRMR
Academic self-regulation	2.44	0.95	0.94	0.95	0.058	0.043
Irrational procrastination scale	2.55	0.97	0.97	0.97	0.050	0.038
Student stress inventory	2.52	0.96	0.95	0.96	0.057	0.050
Academic resilience scale	2.98	0.94	0.94	0.94	0.059	0.049
Academic anxiety	2.77	0.95	0.95	0.95	0.052	0.044

Once the CFAs were conducted, the hypothesized model of predictive relationships was tested on secondary school students (Figure 1), which revealed the following fit indices: χ2 (371. N = 991) = 894.63, *p* < 0.001; χ2/df = 2.41; CFI = 0.96; IFI = 0.96; TLI = 0.96 RMSEA = 0.053 (95% CI = 0.052–0.059); SRMR = 0.041.

The relationships obtained between the different factors comprising the model are described as follows:

(*H1*) Academic self-regulation was negatively related to irrational procrastination (*β* = −0.61, *p* < 0.001).

(*H2*) Irrational procrastination was positively related to academic anxiety (*β* = 0.66, *p* < 0.001) and academic stress (*β* = 0.27, *p* < 0.001).

(*H3*) Academic Resilience was negatively related to academic anxiety (*β* = −0.32, *p* < 0.005) and academic stress (*β* = −0.58, *p* < 0.01).

(*H4*) Academic performance was negatively predicted by academic anxiety (*β* = −0.43, *p* < 0.01) and academic stress (*β* = −0.57, *p* < 0.001), on the other hand, academic performance was positively predicted by academic resilience (*β* = 0.46, *p* < 0.001).

## Discussion

The aim of this study was to analyze students’ behaviors related to academic self-regulation, procrastination, stress, anxiety and academic resilience. These factors were taken into account as determinants toward the predictability of academic performance.

Results have shown that academic self-regulation negatively predicts young people’s procrastination. These results are related to previous studies. In this regard, a study by Durán (2017) found a low negative correlation between academic self-regulation and procrastination in 290 psychology students. Similarly, the study by Bedoya (2017), who reported similar results in a sample of 196 first-year vocational students. Likewise, it has been found that those students with high academic performance showed a high capacity for self-regulation and the use of metacognitive and repetition strategies, which are contrary to procrastination (Wolters et al., 2017).

Furthermore, the results showed that procrastination was positively related to academic stress and anxiety. These results are clearly similar to the study by Vahedi et al. (2012), who found that those students with higher levels of procrastination also had higher levels of academic anxiety, based on a sample of 246 students. Similarly, the study conducted by Pardo et al. (2014), with 100 psychology students from Colombian universities, confirmed that the greater the procrastination, the greater the predicted emotional response of anxiety. In short, it has been consistently shown that procrastination may be associated with factors such as anxiety in the academic environment, in learning and with tension or stress before the next academic tasks are due (Chan, 2011; Clariana et al., 2011). Procrastination has been found to ultimately lead to deterioration or failure in academic skills (Li et al., 2020), as well as stress and physical health (Qian and Fuqiang, 2018), which could ultimately lead to school dropout, hence the importance of working on the prevention of such behaviors.

Finally, resilience would exert a protective role with respect to stress and anxiety, variables with which it is negatively related. In this sense, previous research is along the same lines, finding that those primary and secondary school students with high levels of resilience successfully managed stressful situations (Johnston-Wilder et al., 2014) or the study by Trigueros et al. (2020b), in which it was found that university students with high levels of resilience reported less anxiety before exams. It also seems that academic challenges and challenges would be key in the resilience-building process (Van Hoek et al., 2019). These results are consistent with previous studies indicating that under stressful conditions, the lack and effective use of resilience skills may be revealed (Reich et al., 2010), or, in other words, that stress is negatively associated with resilience (Wilks and Spivey, 2010). It has also been found that more resilient individuals are able to recover earlier from adverse psychoemotional reactions to stress, and are better able to cope with adversity, while showing greater engagement and responsiveness to positive everyday events (Ang et al., 2022).

In turn, the direct role of these three variables (resilience, stress and academic anxiety) on performance has been studied. Resilience, as discussed above, would play a positive role on performance, whereas stress and anxiety would have a negative impact on performance. The proposed model could have important implications for improving academic performance. In this regard, previous research analyzing protective factors for school failure, as well as the impact of risk predictors, found that therapies such as mindfulness reduced students’ stress levels and anxiety (Oblitas et al., 2019), learning to perceive one’s resources to manage adverse situations (Datu and Yuen, 2018) and the use of resilience-related strategies that promote active coping with problems (e.g., problem solving or positive reinterpretation). Thus, the results of this study highlight the importance of promoting resilience in educational settings as it contributes to students’ well-being and quality of life (Seligman and Csikszentmihalyi, 2000). For the effective development of resilience in the individual, he or she must manage the adversity factors related to social disintegration, negative thinking and the absence of effective participation in the events present in his or her life.

The results of the study have a number of implications that we consider to be of interest for the improvement of teaching practice. Firstly, it has been pointed out that it is advisable to promote self-regulation processes in students, and there is a broad consensus among researchers that this can be worked on through repeated instruction and practice in multiple and different contexts. For example, following the proposals of Zimmerman et al. (1996) and Zimmerman and Schunk (2001), it would be desirable to foster psychological processes closely related to self-regulation, such as motivation, goal setting, strategy use and reflection. Following their proposal, through a training model structured in 5 weeks, it is intended that the teacher promotes self-regulatory strategies such as planning and task management, understanding and synthesis of content, writing, note-taking and exam preparation. Self-regulated learners are aware of the role of their activity and personal involvement in their learning process and results, adjusting and monitoring their work, as they are aware of their abilities and skills. In this way, academic self-regulation forms autonomous, independent and self-directed learners (Robson et al., 2020).

On the other hand, it is necessary to analyze what factors might account for procrastination. It may be the case that students intend to finish homework on time, but lack motivation due to dislike or difficulty of the task. Also, sometimes procrastination behavior is reinforced by academic success, which encourages future work-under-pressure behavior. In this sense, the teacher should identify difficulties and encourage the use of strategies such as setting daily goals, planning and establishing a routine, selecting a distraction-free environment that invites concentration, the importance of self-reinforcement and group study. Avoiding procrastination is also about avoiding the stress and anxiety that can be caused by not having enough time to complete an assignment. In this sense, Yu et al. (2021), suggest the need to train teachers in the practice of innovative cognitive learning strategies, encourage decision-making in students, promoting support strategies that promote emotional balance, eliminating fears, as well as, to carry out coexistence between teachers and students that promote an increase in self-esteem.

With regard to academic resilience, the need to incorporate elements focused on emotional regulation, helping to respond to stress, anxiety or other negative emotions, is pointed out. It could be a good initiative to include cross-curricular programes on emotional learning and management and mindfulness.

However, there are a number of limitations to be taken into account: firstly, this is a relational study, which implies the absence of manipulation of variables, so that the causal relationship between the factors studied could go in both directions, and not only in the one indicated in the proposed model. In this sense, it would be interesting to replicate this study using a longitudinal methodology that would allow us to confirm the proposed relationships and the specific weight of each variable in subsequent academic performance. It is also convenient to take into consideration the small sample size and the non-random sampling, so it would be interesting to observe what happens in a larger sample obtained by random sampling. Nevertheless, despite the limitations mentioned above, the model seems to show good robustness and generalisability.

## Data availability statement

The data analyzed in this study is subject to the following licenses/restrictions: We do not have the permission of the participants to disclose the database. Requests to access these datasets should be directed to RT, rtr088@ual.es.

## Ethics statement

The studies involving human participants were reviewed and approved by REF: UAL/BIO 213/2020. Written informed consent to participate in this study was provided by the participants’ legal guardian/next of kin.

## Author contributions

All authors listed have made a substantial, direct, and intellectual contribution to the work, and approved it for publication.

## Conflict of interest

The authors declare that the research was conducted in the absence of any commercial or financial relationships that could be construed as a potential conflict of interest.

## Publisher’s note

All claims expressed in this article are solely those of the authors and do not necessarily represent those of their affiliated organizations, or those of the publisher, the editors and the reviewers. Any product that may be evaluated in this article, or claim that may be made by its manufacturer, is not guaranteed or endorsed by the publisher.
